# Tpz1^TPP^^1^ SUMOylation reveals evolutionary conservation of SUMO-dependent Stn1 telomere association

**DOI:** 10.15252/embr.201438919

**Published:** 2014-06-12

**Authors:** Mansi Garg, Resham L Gurung, Sahar Mansoubi, Jubed O Ahmed, Anoushka Davé, Felicity Z Watts, Alessandro Bianchi

**Affiliations:** Genome Damage and Stability Centre, School of Life Sciences, University of SussexBrighton, UK

**Keywords:** CST, Stn1, telomeres, Tpz1, SUMO

## Abstract

Elongation of the telomeric overhang by telomerase is counteracted by synthesis of the complementary strand by the CST complex, CTC1(Cdc13)/Stn1/Ten1. Interaction of budding yeast Stn1 with overhang-binding Cdc13 is increased by Cdc13 SUMOylation. Human and fission yeast CST instead interact with overhang-binding TPP1/POT1. We show that the fission yeast TPP1 ortholog, Tpz1, is SUMOylated. Tpz1 SUMOylation restricts telomere elongation and promotes Stn1/Ten1 telomere association, and a SUMO-Tpz1 fusion protein has increased affinity for Stn1. Our data suggest that SUMO inhibits telomerase through stimulation of Stn1/Ten1 action by Tpz1, highlighting the evolutionary conservation of the regulation of CST function by SUMOylation.

## Introduction

Telomeres, nucleoprotein complexes assembled at chromosome ends and maintained through the ability of the telomerase enzyme to synthesise telomeric DNA repeats, preserve genome stability by preventing the activation of DNA damage checkpoints and DNA repair pathways. The action of telomerase at telomeres is finely regulated in a complex manner by a plethora of protein factors bound to the DNA repeats 1,2. Several of these factors, particularly those that are assembled on the double-stranded part of telomeres, act as negative regulators of the enzyme while others, primarily associated with the terminal single-stranded overhang, are required for telomerase activity and function in promoting either its recruitment or its activity at ends. In human cells, the core telomeric complex is made of six proteins collectively known as shelterin 3. Within shelterin, TRF1 and TRF2 contact double-stranded DNA, recruiting RAP1 and TIN2, and the latter protein binds TPP1 which in turn interacts with overhang-binding POT1. While TRF1/TRF2/RAP1 are inhibitory of telomerase action, POT1 and TPP1 act to stimulate telomerase activity *in vitro*, and TPP1, via its interaction with TIN2, is directly involved in recruiting telomerase to telomeres 4. This telomere architecture has a close parallel in fission yeast, where Taz1^TRF^ and Rap1 restrain telomerase action together with Poz1, which, like TIN2, acts as a molecular link to the overhang-bound Tpz1^TPP1^/Pot1 5–7. In fission yeast, Tpz1 mediates telomerase recruitment via its interaction with Ccq1 6,8. In addition to shelterin, an additional complex, CST, made of the three subunits CTC1(Cdc13)/STN1/TEN1 and with structural similarities to single-stranded DNA binding protein RPA, has a crucial role in telomere protection and the control of telomerase activity 9–12. In both these processes, the function of CST is partly in promoting the synthesis of the telomeric lagging strand, which is thought to constrain telomerase activity by reducing the availability of the single-stranded DNA substrate 13–18.

The interplay of this set of proteins in modulating telomerase activity and telomere length is incompletely understood, but it is clear that post-translational modifications of telomeric proteins play an important role. For example, the ATM and ATR kinase homologs are required in both fission and budding yeast to guarantee telomerase action: in fission yeast, Rad3^ATR^ and Tel1^ATM^ phosphorylate Ccq1 to allow its interaction with Est1 and telomerase 19,20. SUMOylation, in addition to phosphorylation, has also been implicated in controlling telomerase. In budding yeast, SUMOylation of Cdc13 (which is utilised as the main overhang-binding factor in this organism devoid of Pot1) has been shown to inhibit telomerase by stimulating the interaction of Cdc13 with Stn1 21. In fission yeast, previous work has indicated that telomerase activity is similarly kept in check by the SUMO pathway 22,23.

## Results and Discussion

### Fission yeast Tpz1 is SUMOylated at lysine 242

**Figure 1:**
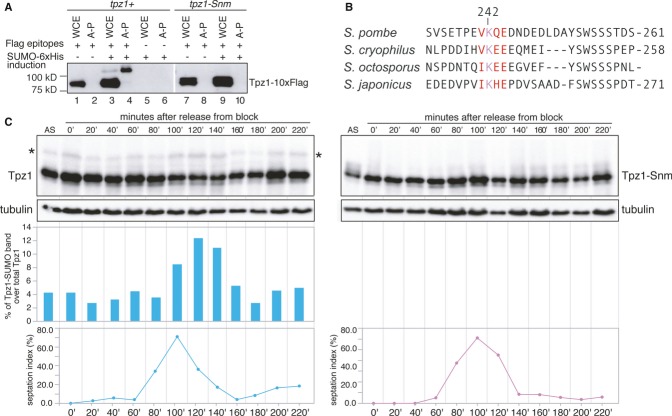
Tpz1 is SUMOylated at lysine 242 A Affinity-purification of SUMO targets. Fission yeast cells contained a 10x-Flag epitope tag at the Tpz1 C-terminus and a plasmid expressing a 6x-histidine tagged version of SUMO (Pmt3) under control of the *nmt41* promoter, which is repressed by thiamine and slowly induced upon thiamine withdrawal. At 22 h after thiamine removal, whole-cell protein extracts (WCE) were prepared. SUMO-modified proteins were then affinity-purified (A-P) on Ni-agarose beads under denaturing conditions. Both whole-cell extract and affinity-purified material were analysed by Western blotting with anti-Flag M2 antibody. B Sequence alignment of the indicated portions of the Tpz1 proteins from the four sequenced *Schizosaccharomyces* species. The four-residue SUMOylation consensus is in red with the target lysine in purple. C Cell cycle regulation of Tpz1 SUMOylation. Western analysis of WCEs prepared from Tpz1-10xFlag cells otherwise wild-type (left panel) or *tpz1-Snm* (right panel), also carrying the *cdc25*-*22* temperature-sensitive allele. ‘AS’ indicates asynchronous cultures, whereas the other lanes show samples taken at the indicated times (at 25°C) after release from cell cycle arrest at the restrictive temperature. The asterisks indicate the SUMOylated Tpz1 band. The middle panel shows the quantification of the Tpz1 SUMOylated species from the gel directly above, as a percentage of the total Tpz1 signal normalised against a tubulin loading control. The bottom panels indicate the septation index (a marker of S phase) for the samples directly above.

To investigate the role of SUMO as a regulator of telomere function, we sought to determine whether any of the core components of the fission yeast telomeric complex are post-translationally modified by SUMOylation. Into several fission yeast strains, each carrying a flag-tagged version of a telomeric protein, we introduced a 6-histidine tagged version of the SUMO protein (the product of the *pmt3^+^* gene) put under control of an inducible promoter, for affinity-purification of SUMOylated proteins under denaturing conditions expected to destabilise non-covalent protein linkage 24. This analysis revealed the presence of a band in the affinity-purified material in strains containing a Flag-tagged version of Tpz1 (Fig 1A, lane 4; compare to lane 6). This band was present only upon induction of expression of the modified SUMO gene (Fig 1A, compare lanes 2 and 4), suggesting that it might represent SUMOylated Tpz1. Importantly, the migration rate of this protein species was slower compared to that of unmodified Tpz1, consistent with addition of SUMO to Tpz1.

An analysis of potential sites of SUMO-modification in *S. pombe* Tpz1 25 uncovered a unique site conforming to the ψKx(D/E) consensus (where ‘ψ’ is any large hydrophobic residue and ‘x’ is any amino acid), overlapping lysine 242 (Fig 1B). Significantly, a potential SUMOylation site was also identified in the three other *Schizosaccharomyces* species whose genome has been sequenced, at essentially the same position, even though the conservation of the primary sequence in this region is poor. We therefore proceeded to create *S. pombe* strains bearing a conservative lysine to arginine change at position 242. In strains with a Flag-tagged copy of the *tpz1-K242R* allele expressing the 6x-histidine SUMO variant, we were unable to recover Tpz1 in the affinity-purified fractions, contrary to wild-type (Fig 1A, compare lane 10 to lane 4), indicating that lysine 242 is required for covalent modification of Tpz1 by SUMO. As a consequence, we renamed the *tpz1-K242R* allele as *tpz1-Snm* (SUMO no more) 21.

Analysis of Tpz1 in the absence of the histidine-modified *Pnmt*-driven SUMO indicated the presence of a slowly migrating species, of similar size to the 6x-histidine-SUMO-Tpz1 protein, suggesting that SUMOylation of Tpz1 takes place under normal conditions of SUMO expression (Fig 1C, top, left panel, AS lane). The identity of this slowly migrating Tpz1 as SUMOylated protein was supported by the fact that this band was absent in protein extracts obtained from *tpz1-Snm* cells (Fig 1C, top, right panel). Because of the potential regulatory role of the modification, we conducted this analysis in synchronous cultures to determine whether SUMOylation levels of Tpz1 vary during the cell cycle. Previous studies of budding yeast Cdc13 indicated that SUMOylation of this protein factor raise during S phase 21. Similarly, we found that SUMOylation of Tpz1 peaked during late S phase, the time of telomere replication and telomerase action, suggesting a possible regulatory role for the modification (Fig 1C, middle panel).

### SUMOylation of Tpz1 limits telomere elongation

**Figure 2:**
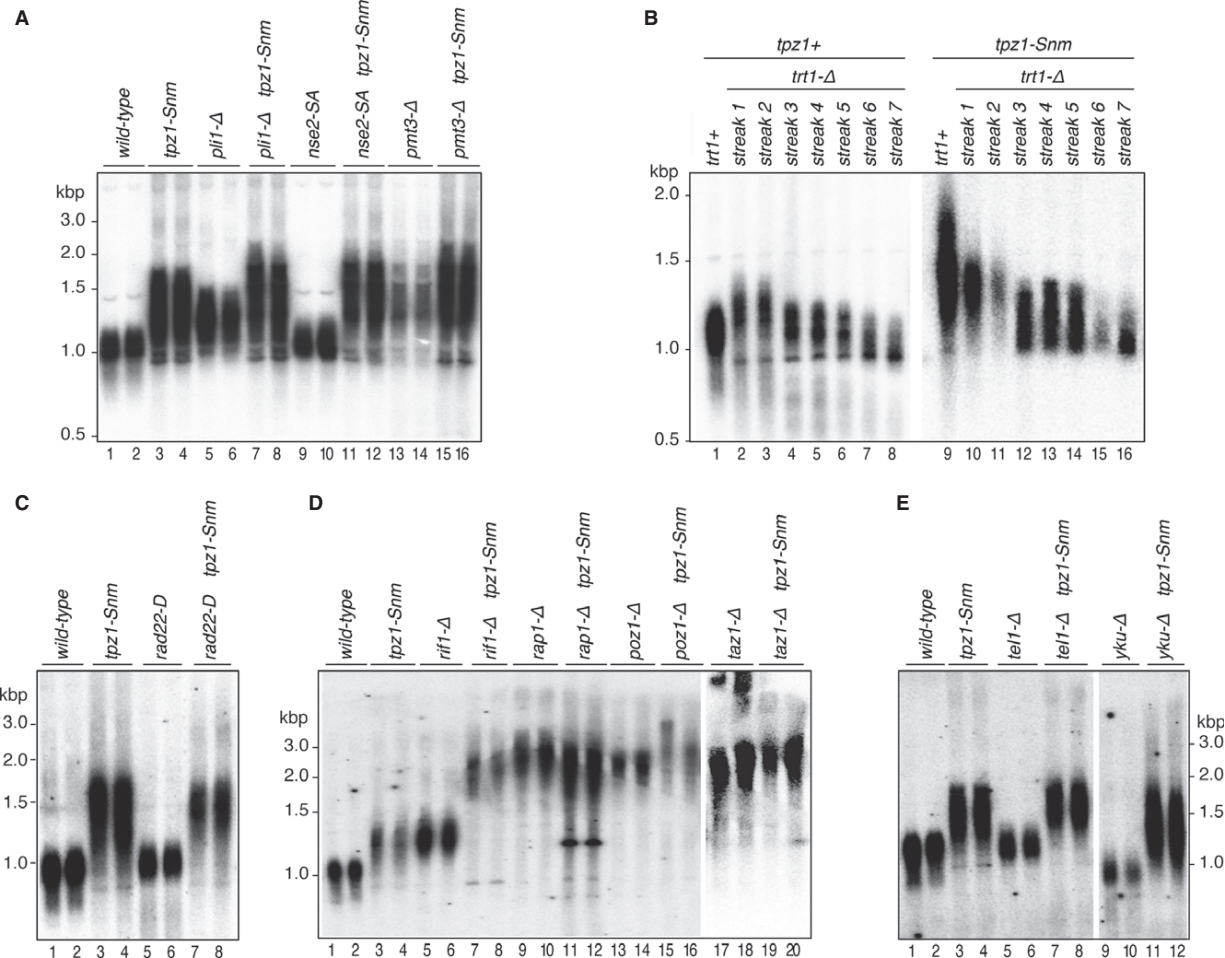
Tpz1 SUMOylation down-regulates telomerase action Analysis of telomere length in various mutant backgrounds. A–E Genomic DNA from fission yeast cells of the indicated genotypes was prepared and analysed by Southern blotting with a radiolabelled telomeric DNA probe. The *nse2-SA* allele carries two mutations in the RING domain that abolish ligase activity. In panel B, the strains in lanes 2–8 and 10–16 carried a deletion of the *trt1*^+^ gene; the deletion was complemented by a plasmid-borne wild-type copy of the *trt1^+^* and the samples represents serial culturing of this strain after loss of the plasmid (which carries an *ade6*^+^ and a *Padh1*-driven *tk* gene) achieved by growth (in streak 1) in FuDR-containing medium.

As observed previously 22,23, loss of the E3 Pli1 ligase led to telomere elongation, whereas mutation of the second known E3 ligase, Nse2, did not, and loss of Pmt3 conferred longer telomeres compared to loss of Pli1 (Fig 2A, lanes 5, 6, 9, 10, 13, 14). Strikingly, the *tpz1-Snm* allele also led to telomere elongation, almost to the same extent as loss of SUMO itself (Fig 2A, compare lanes 3, 4 and 13, 14), indicating that modification of lysine 242 of Tpz1 is primarily responsible for the effect of SUMO on telomere length. As expected, analysis of the telomere length in double mutants indicated that *tpz1-Snm* is epistatic to mutations of the SUMO E3 ligases or SUMO itself (Fig 2A, lanes 7,8,11,12,15,16). In addition, the relatively small difference in telomere length between *pli1-Δ* and *tpz1-Snm* cells suggests that Pli1 is the E3 ligase primarily responsible for the modification of Tpz1.

Fission yeast telomeres, like those of other species, can be maintained by either telomerase action or by recombination. To test whether either one of these telomere maintenance pathways, or possibly both, contributed to telomere lengthening in *tpz1-Snm* cells, we introduced the mutation in cells bearing their functional copy of telomerase on a counter-selectable plasmid. After plasmid loss, the telomerase-deficient *tpz1-Snm* cells underwent progressive telomere shortening, indicating that telomerase acted downstream of Tpz1 SUMOylation (Fig 2B). In other words, modification of K242 in Tpz1 by SUMO affects telomere length in a telomerase-dependent manner. Instead, recombination is not required for the telomere length phenotype of the *tpz1-Snm* allele nor does it make a significant contribution to it, as judged by epistasis analysis with a *rad22* null allele, which impairs homologous recombination (Fig 2C). These results are consistent with earlier findings with *pli1* 23.

To begin to address the mechanism of telomerase regulation by Tpz1 SUMOylation, we combined the *tpz1-Snm* allele with several null mutations known to affect telomerase action. Among negative regulators of telomerase, mutation of *rif1* clearly displayed a synergistic effect with *tpz1-Snm*, indicating that the two alleles affect different pathways (Fig 2D, lanes 3–8). Deletion of the genes in this group which are part of the shelterin-like complex in fission yeast (*rap1, poz1* and *taz1*) maintained very long telomeres in the presence of *tpz1-Snm*, suggesting that they act downstream of SUMOylation in the control of telomere length (Fig 2D, lanes 9–20). For *rap1*, telomeres actually appeared slightly shorter in the double mutant, suggesting that in the absence of Rap1, SUMOylated Tpz1 might act to promote telomere elongation, consistent with the telomere length phenotype of *rap1 pli1* cells 23. Neither loss of *tel1* or *yku* (Fig 2D, lanes 5–12) prevented telomere elongation by *tpz1-Snm,* suggesting that the latter allele largely acts downstream of their action.

### Tpz1 SUMOylation is required for telomere association of Stn1/Ten1

**Figure 3:**
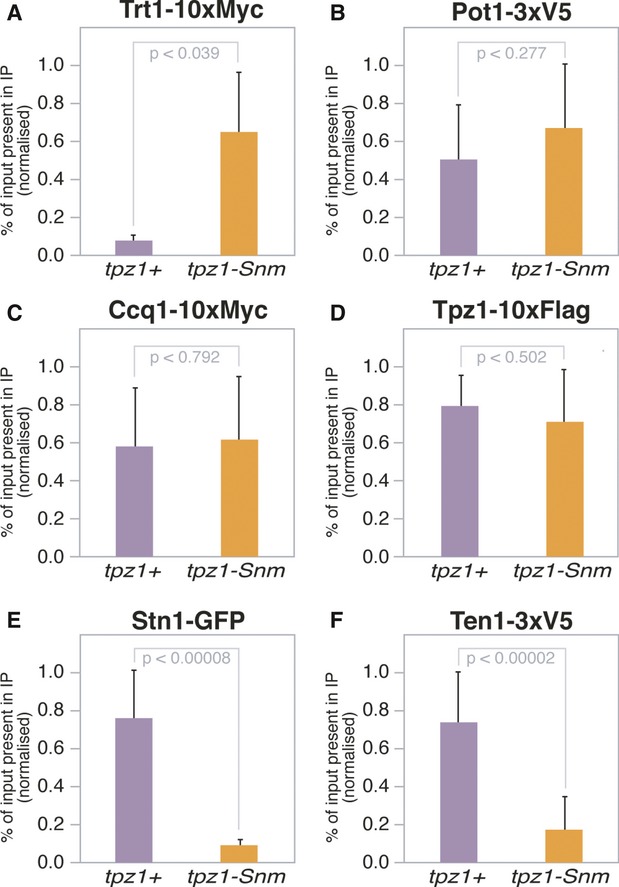
Tpz1 SUMOylation is required for efficient recruitment of Stn1/Ten1 to telomeres A–F Association of the indicated epitope-tagged proteins with telomeres was determined as described under Materials and Methods using dot-blot hybridisation quantification. Histograms represent the averages of 3, 9, 6, 7, 6 and 12 replicates, respectively, obtained from 1, 3, 2, 3, 2 and 4 independent experiments (for A, an independent experiment using a 3V5 tag also yielded a significant difference in association, *P* < 0.020). Error bars are standard deviations, and *P*-values are calculated from two-tailed *t*-tests.

To better understand the mechanism of action of Tpz1-SUMO in the telomerase pathway, we turned to chromatin immunoprecipitation (ChIP), reasoning that telomeric SUMO might modulate the affinity/recruitment of telomeric proteins to telomeres. We compared the telomere association of several proteins in wild-type *tpz1^+^* and *tpz1-Snm* cells. This analysis identified a significant increase in telomere binding for telomerase in the mutant (Fig 3A), which can account for the observed increase in telomere length in these cells, but not for the Tpz1-binding partners Pot1 and Ccq1, or for Tpz1 itself (Fig 3B–D). On the contrary, the two members of the CST complex that are present in *S. pombe*, Stn1 and Ten1 26 showed a prominent reduction in telomere binding in the mutant compared to wild-type (Fig 3E and F). These results indicate that SUMOylation of Tpz1 does not induce gross changes in the shelterin-like complex, but rather acts in promoting the recruitment of Stn1/Ten1 to telomeres.

### Interaction between SUMOylated Tpz1 and Stn1

**Figure 4:**
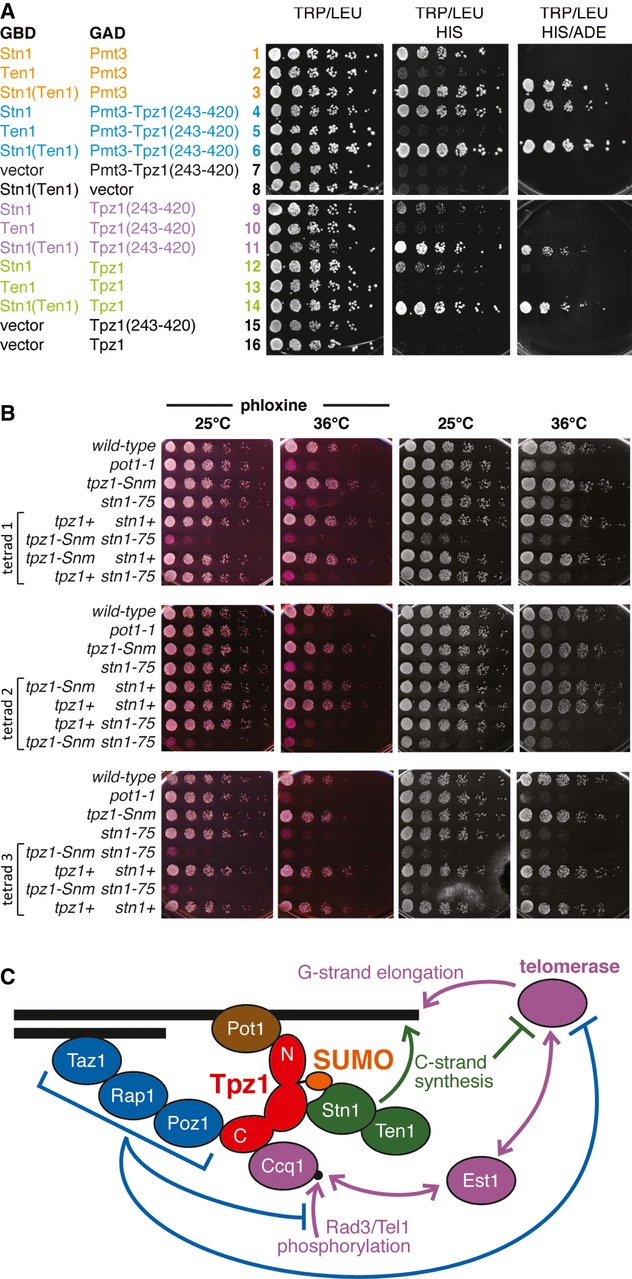
SUMO interacts with Stn1 A Yeast two-hybrid analysis of budding yeast strains carrying the indicated plasmids with fission yeast proteins fused to either Gal4 DNA binding (GBD) or Gal4 activating domain (GAD). Strains were grown in liquid and then spotted in fivefold serial dilutions on the indicated plates selecting for plasmids (TRP and LEU) or for activation of one (HIS) or two reporters (HIS and ADE). Plates were allowed to grow at 30°C for 2 days. B Analysis of viability of indicated strains at different temperatures. Cultures of strains at 25°C in the exponential phase were serially diluted (fivefold) and plated on rich medium (YES), containing (left), or not (right), phloxine to highlight poorly growing cells, which do not export the dye efficiently and are therefore darker in colour. Three tetrads from the sporulation of a *tpz1-Snm stn1-75* heterozygous diploid were sporulated. The *stn1-75* allele has a single amino acid change, L198S. C Diagram representing some of the key events in telomerase regulation in fission yeast. Double arrows indicate physical interactions. For Tpz1, the N- and C-termini are indicated; both the Tpz1 central domain (amino acids 243–420) and its covalently linked SUMO moiety (at position 242) are proposed to directly interact with Stn1.

Because SUMO is known to modulate biological processes by providing a binding surface for proteins that have the ability to bind to it, we considered the possibility that our ChIP results could be explained by a role of SUMO in enhancing a direct interaction between Tpz1 and Stn1/Ten1 27. To test this idea, we conducted a series of yeast two-hybrid assays, using a strain that allowed us to monitor the activation of a HIS3 and a (more stringent) ADE2 reporter. Under the less stringent conditions (HIS selection), we were able to detect a weak interaction between full-length Stn1 and Tpz1 proteins, but not between Ten1 and Tpz1 (Fig 4A, rows 12 and 13). Although co-expression of Ten1 alongside the GBD-Stn1 fusion protein increased the strength of the interaction, now visible on plates lacking adenine as previously reported 27 (row 14), the data indicated that Stn1 is capable of interacting directly with Tpz1. Whether Ten1 associates with Stn1 to form direct protein contacts with Tpz1, or it might simply bind Stn1 and thus contribute to its stability and/or folding, is unclear. Previous work identified the region of Tpz1 between amino acids 224 and 420 as being minimally required for the binding of Tpz1 to Stn1/Ten1 27. Because this domain overlaps K242, we investigated whether SUMO and a subdomain of Tpz1 can together constitute a Tpz1-interacting domain of higher affinity for Stn1. We first established that a Tpz1 domain truncated at the SUMOylation site (243–420) was capable of interaction with Stn1 to a level comparable to full-length Tpz1, either in the absence or presence of Ten1 (compare rows 9 and 12; and 11 and 14 in Fig 4). We then assessed the ability of SUMO (Pmt3) to bind Stn1 and found that the proteins do interact (Fig 4, row 1). Ten1, which on its own did not bind SUMO significantly (Fig 4, row 2), was able to strongly stimulate the interaction between Stn1 and SUMO (Fig 4, row 3), as it did for Stn1 and Tpz1. Interestingly, when SUMO was fused to the N-terminus of the Tpz1 243–420 domain, thus mimicking the naturally occurring SUMOylated Tpz1 protein, the interaction with Stn1 was enhanced, with respect to both the Tpz1 243–420 and SUMO proteins alone (Fig 4, compare row 4 with 9 and 1, respectively). The same was observed in the presence of Ten1 (Fig 4, compare row 6 with 11 and 3): the SUMO-Tpz1(243–420) construct in the presence of Ten1 conferred the strongest observed growth in the Stn1 interaction assays. Taken together, these results indicate that a central domain of Tpz1 encompassing amino acids 243–420 is sufficient to promote an interaction with Stn1 and that this interaction is stabilised by Ten1. In addition, SUMO is capable of interacting with Stn1 independently, and linkage of SUMO at its naturally occurring site in Tpz1 strongly enhances the ability of Tpz1 to interact with Stn1. This interaction can explain the effect of *tpz1-Snm* on the telomere association of Stn1/Ten1 (Fig 3E and F), and its significance is consistent with the increased level of the modification observed in late S phase (Fig 1C) concomitant with the observed time of recruitment of Stn1/Ten1 to telomeres 28.

Both the ChIP and yeast two-hybrid analysis strongly indicate that the function of Stn1 at fission yeast telomeres is modulated by SUMOylation. Because both methods assess physical interactions, within the telomeric complex and between proteins, we sought independent genetic evidence for this idea. We performed random-mutagenesis on *stn1^+^*, and produced an allele, *stn1-75*, which is defective for *stn1* function at 36°C as judged by colonies growing poorly and turning deep red on phloxine plates at this temperature, indicative of likely telomere loss (Fig 4B, fourth row in each of the three groups of plates). The *pot1-1* allele, which is also non-functional at 36°C and leads to telomere loss and chromosome circularisation, was used as a control 29. While the *tpz1-Snm* allele on its own did not confer any growth defect to cells (Fig 4, third row), sporulation of a heterozygous *tpz1-Snm stn1-75* diploid (Fig 4B, tetrads 1–3) revealed that, remarkably, *tpz1-Snm* affected the viability of *stn1-75* cells at 25°C, contrary to wild-type *tpz1^+^* and consistent with the idea that SUMOylation of Tpz1 facilitates the telomere recruitment—and hence the function—of Stn1. In Fig 4C, we summarise the molecular interactions promoted by SUMOylated Tpz1 at telomeres as suggested by our findings.

## Conclusions

The CST complex, originally suspected to be important primarily at budding yeast telomeres, has in recent years been recognised as having a widespread role in telomere protection and the regulation of telomerase action in a number of species 11,12,30. The emerging picture, from studies both in yeast and in mammalian cells, is that CST is required to promote synthesis of the C-strand by the lagging strand DNA replication machinery likely after telomerase action on the G-strand 13–18,31. Consistent with its role in completing DNA replication, yeast Stn1 associates with telomeres preferentially late in S phase 15,28. In budding yeast, which lacks Pot1 and instead utilises Cdc13 as the main overhang-binding activity, it has been suggested that Stn1/Ten1 compete with Est1 for interaction with Cdc13 and that the outcome of this competition is governed by independent modifications of Cdc13, with phosphorylation by CDK favouring Est1 binding, and SUMOylation instead promoting Stn1/Ten1 association 21. In mammalian cells, various interactions have been reported between CST (in particular STN1) and the overhang-binding heterodimer POT1/TPP1 16,18,32, similar to fission yeast 27. Overall, these studies suggest that Stn1/Ten1 require an association with core telomeric components for recruitment. Our data, together with a similar recently published study 33, reveal a remarkable conservation in the mechanism of this recruitment by showing that Stn1 relies on SUMOylation of a telomere protein for association with telomeres, raising the obvious possibility that mammalian STN1 might similarly rely on a SUMOylated telomeric binding partner.

## Materials and Methods

### Strains and plasmids

Culture conditions and a complete list of fission yeast strains are reported in Supplementary Table S1. C-terminal epitope-tagging of various proteins was carried using plasmids containing the protein’s C-terminus cloned in-frame to appropriate epitope tags linked by an 8xGlycine linker. A list of the plasmids used is reported in Supplementary Table S2.

### Telomere length analysis by Southern blotting

Genomic DNA was prepared from log-phase cultures, digested with Eco RI and electrophoresed on 1% agarose gels. Hybridisation was carried out with a radiolabelled telomere probe.

### Chromatin immunoprecipitation

For ChIP quantification, DNA samples from WCEs and IPs were spotted to a nylon membrane for hybridisation using a radiolabelled telomeric DNA probe.

### Two-hybrid analysis

Yeast two-hybrid assays were performed by co-transforming Gal4 DNA binding domain (GBD) and Gal4 activating domain (GAD) plasmids in various combinations in the PJ69-4A budding yeast strain (*MATa trp1-901 leu2-3,112 ura3-52 his3-200 gal4 gal80 LYS2::GAL1-HIS3 GAL2-ADE2 met2::GAL7-lacZ)*. Transformants were screened for interaction by spotting fivefold dilutions on selective Sc-TRP-LEU-HIS and Sc-TRP-LEU-HIS-ADE media.

## References

[b1] Shore D, Bianchi A (2009). Telomere length regulation: coupling DNA end processing to feedback regulation of telomerase. EMBO J.

[b2] Jain D, Cooper JP (2010). Telomeric strategies: means to an end. Annu Rev Genet.

[b3] Palm W, de Lange T (2008). How shelterin protects mammalian telomeres. Annu Rev Genet.

[b4] Abreu E, Aritonovska E, Reichenbach P, Cristofari G, Culp B, Terns RM, Lingner J, Terns MP (2010). TIN2-tethered TPP1 recruits human telomerase to telomeres in vivo. Mol Cell Biol.

[b5] Cooper JP, Nimmo ER, Allshire RC, Cech TR (1997). Regulation of telomere length and function by a Myb-domain protein in fission yeast. Nature.

[b6] Miyoshi T, Kanoh J, Saito M, Ishikawa F (2008). Fission yeast Pot1-Tpp1 protects telomeres and regulates telomere length. Science.

[b7] Jun HI, Liu J, Jeong H, Kim JK, Qiao F (2013). Tpz1 controls a telomerase-nonextendible telomeric state and coordinates switching to an extendible state via Ccq1. Genes Dev.

[b8] Tomita K, Cooper JP (2008). Fission yeast Ccq1 is telomerase recruiter and local checkpoint controller. Genes Dev.

[b9] Sun J, Yu EY, Yang Y, Confer LA, Sun SH, Wan K, Lue NF, Lei M (2009). Stn1-Ten1 is an Rpa2-Rpa3-like complex at telomeres. Genes Dev.

[b10] Gao H, Cervantes RB, Mandell EK, Otero JH, Lundblad V (2007). RPA-like proteins mediate yeast telomere function. Nat Struct Mol Biol.

[b11] Miyake Y, Nakamura M, Nabetani A, Shimamura S, Tamura M, Yonehara S, Saito M, Ishikawa F (2009). RPA-like mammalian Ctc1-Stn1-Ten1 complex binds to single-stranded DNA and protects telomeres independently of the Pot1 pathway. Mol Cell.

[b12] Surovtseva YV, Churikov D, Boltz KA, Song X, Lamb JC, Warrington R, Leehy K, Heacock M, Price CM, Shippen DE (2009). Conserved telomere maintenance component 1 interacts with STN1 and maintains chromosome ends in higher eukaryotes. Mol Cell.

[b13] Chandra A, Hughes TR, Nugent CI, Lundblad V (2001). Cdc13 both positively and negatively regulates telomere replication. Genes Dev.

[b14] Qi H, Zakian VA (2000). The *Saccharomyces* telomere-binding protein Cdc13p interacts with both the catalytic subunit of DNA polymerase alpha and the telomerase- associated est1 protein. Genes Dev.

[b15] Puglisi A, Bianchi A, Lemmens L, Damay P, Shore D (2008). Distinct roles for yeast Stn1 in telomere capping and telomerase inhibition. EMBO J.

[b16] Wang F, Stewart JA, Kasbek C, Zhao Y, Wright WE, Price CM (2012). Human CST has independent functions during telomere duplex replication and C-strand fill-in. Cell Rep.

[b17] Chen LY, Redon S, Lingner J (2012). The human CST complex is a terminator of telomerase activity. Nature.

[b18] Wu P, Takai H, de Lange T (2012). Telomeric 3’ overhangs derive from resection by Exo1 and Apollo and fill-in by POT1b-associated CST. Cell.

[b19] Yamazaki H, Tarumoto Y, Ishikawa F (2012). Tel1(ATM) and Rad3(ATR) phosphorylate the telomere protein Ccq1 to recruit telomerase and elongate telomeres in fission yeast. Genes Dev.

[b20] Moser BA, Chang YT, Kosti J, Nakamura TM (2011). Tel1(ATM) and Rad3(ATR) kinases promote Ccq1-Est1 interaction to maintain telomeres in fission yeast. Nat Struct Mol Biol.

[b21] Hang LE, Liu X, Cheung I, Yang Y, Zhao X (2011). SUMOylation regulates telomere length homeostasis by targeting Cdc13. Nat Struct Mol Biol.

[b22] Xhemalce B, Seeler JS, Thon G, Dejean A, Arcangioli B (2004). Role of the fission yeast SUMO E3 ligase Pli1p in centromere and telomere maintenance. EMBO J.

[b23] Xhemalce B, Riising EM, Baumann P, Dejean A, Arcangioli B, Seeler JS (2007). Role of SUMO in the dynamics of telomere maintenance in fission yeast. Proc Natl Acad Sci U S A.

[b24] Ulrich HD, Davies AA (2009). In vivo detection and characterization of sumoylation targets in *Saccharomyces cerevisiae*. Methods Mol Biol.

[b25] Ren J, Gao X, Jin C, Zhu M, Wang X, Shaw A, Wen L, Yao X, Xue Y (2009). Systematic study of protein sumoylation: development of a site-specific predictor of SUMOsp 2.0. Proteomics.

[b26] Martin V, Du LL, Rozenzhak S, Russell P (2007). Protection of telomeres by a conserved Stn1-Ten1 complex. Proc Natl Acad Sci U S A.

[b27] Chang Y-T, Lingner J, Moser BA, Nakamura TM (2013). Fission yeast shelterin regulates DNA polymerases and Rad3ATR kinase to limit telomere extension. PLoS Genet.

[b28] Moser BA, Subramanian L, Chang YT, Noguchi C, Noguchi E, Nakamura TM (2009). Differential arrival of leading and lagging strand DNA polymerases at fission yeast telomeres. EMBO J.

[b29] Pitt CW, Cooper JP (2010). Pot1 inactivation leads to rampant telomere resection and loss in one cell cycle. Nucleic Acids Res.

[b30] Giraud-Panis MJ, Teixeira MT, Geli V, Gilson E (2010). CST meets shelterin to keep telomeres in check. Mol Cell.

[b31] Nakaoka H, Nishiyama A, Saito M, Ishikawa F (2012). *Xenopus laevis* Ctc1-Stn1-Ten1 (xCST) protein complex is involved in priming DNA synthesis on single-stranded DNA template in *Xenopus* egg extract. J Biol Chem.

[b32] Wan M, Qin J, Songyang Z, Liu D (2009). OB fold-containing protein 1 (OBFC1), a human homolog of yeast Stn1, associates with TPP1 and is implicated in telomere length regulation. J Biol Chem.

[b33] Miyagawa K, Low RS, Santosa V, Tsuji H, Moser BA, Fujisawa S, Harland JL, Raguimova ON, Go A, Ueno M (2014). SUMOylation regulates telomere length by targeting the shelterin subunit Tpz1T pp1 to modulate shelterin-Stn1 interaction in fission yeast. Proc Natl Acad Sci U S A.

