# Supplementary material for: Tpz1TPP1 SUMOylation reveals evolutionary conservation of SUMO-dependent Stn1 telomere association
Source: EMBO Rep. 2014 Jun 12;15(8):871–7. doi: 10.15252/embr.201438919 (PMC4197044; doi:10.15252/embr.201438919)
Supplement: Supplementary file 3 [file embr0015-0871-sd3.pdf]

Supplementary Materials and Methods

Strains and plasmids

Fission yeast strains were grown in YES rich medium or YNG minimal medium (2% w/v glucose, 30 mM glutamate, 0.17% w/v YNB mix without amino acids, ammonium sulphate or thiamine, 0.53% w/v SC dropout mix, 2% w/v agar, pH 6.0). YNB and SC (synthetic complete) mixes were from United States Biological. For transformations, cells were incubated with plasmid DNA, carrier DNA and 40% PEG/LiAc/TE solution for 3 hrs at 30°C, heat shocked at 42°C for 10 min, and plated out without centrifugation.

C-terminal epitope-tagging of various protein was carried out by modification of the chromosomal loci using linearized plasmids containing the protein's C-terminus cloned in-frame to appropriate epitope tags linked by an 8xGlycine linker.

To obtain the *tpz1-K242R* (*tpz1-Snm*) allele, an NdeI-linearized plasmid (pAB1623) with a *tpz1* fragment containing the mutated region and a *ura+* marker was transformed into yeast. The resultant colonies were counter-selected on 5'-fluoroorotic acid (5'-FOA) to select for recombination at the Sp *tpz1* locus. The presence of the K242R mutation was verified by PCR with DO1497/DO1826 (819 bp product) followed by restriction enzyme digest with HaeIII (429 + 390 bp in *tpz1*-Snm; uncut in WT).

Affinity-purification of SUMOylated Tpz1

Fission yeast cells were grown to a cell density of 1 x 10^7 cells/ml in 110 ml YNG medium lacking leucine and containing 15 mM thiamine. Of these, 100 and 5 ml were centrifuged for 5 min at 4°C at 3000 rpm, and the cell pellets were stored at -20°C as the uninduced fractions (for affinity-purification and WCE preparation, respectively). Cells from the remaining 5 ml cultures were harvested separately and the cell pellets were washed twice with sterile distilled water before resuspension in 110 ml fresh YNG medium lacking leucine with no thiamine. These cultures were then grown for 22 hrs to a cell density of 1 x 10^7 cells/ml to allow 6xhis-SUMO expression. After

22hrs the cells were harvested as done previously and stored at -20˚C, this time, as the induced fraction.

Ni-NTA affinity-purification was performed as described previously [1]. Harvested cells (from the cell pellets derived from the 100 ml cultures) were resuspended in 5 ml ice-cold distilled water followed by the addition of 0.8 ml of freshly prepared NaOH/BME (1.85 M NaOH, 7.5 mM β-mercaptoethanol). After incubating the tubes on ice for 20 min, 0.8 ml of 55% TCA was added, and the tubes were incubated on ice for a further 20 min. Cells were then pelleted by centrifugation at 8000 rpm for 20 min at 4°C. Cell pellets were resuspended in 1 ml of Buffer A (6 M guanidine HCl, 100 mM sodium phosphate pH 8.0, 10 mM Tris-HCl pH 8) and rotated for 1 hour at room temperature. Supernatants were collected by centrifuging the tubes at 16000g for 10 min at 4°C. Ni-NTA agarose beads (Qiagen) were prepared by washing it three times with Buffer A containing 0.05% Tween-20. 20 µl of washed beads were added to the supernatant along with 0.05% Tween-20 and 15 mM imidazole. The final mixtures were incubated at room temperature on a rotating wheel overnight. Then, the tubes were spun at 200 rpm for 15 sec and supernatants were discarded. The beads were washed twice with Buffer A plus 0.05% Tween-20, and washed four times with Buffer C (8 M urea, 100 mM sodium phosphate pH 6.3, 10 mM Tris-HCl pH 6.3) plus 0.05% Tween-20. 30 µl HU buffer (8 M urea, 200 mM Tris-HCl pH 6.8, 1 mM EDTA, 5% SDS, 0.1% bromophenol blue, 1.5% DTT) were added to the agarose beads. To prepare WCEs, pelleted cells were resuspended in 500 µl of distilled water followed by the addition of 75 µl of freshly prepared NaOH/BME and were incubated on ice for 15 min. 75 µl 55% TCA was added and the tubes were incubated on ice for a further 10 min. The lysates were centrifuged at 16000 rpm for 10 min at 4°C and the pellets were resuspended in 60 µl HU buffer. Both affinity-purified proteins and WCEs were heated at 60°C for 10 min before being analysed by SDS-PAGE.

Analysis of Tpz1 SUMOylation levels

Fission yeast strains containing the *cdc25-22* allele were grown to mid-log-phase at 25°C, arrested in G2 for 3 hrs at 36°C and subsequently released at 25°C for 220 min. Samples were collected every 20 min after release and the septation index was determined. Cells were

resuspended in 600 µl 100%TCA, kept on ice for 10 min and then pelleted by centrifuging at 3000 rpm for 2 min, followed by two acetone washes. The pellets were then dried under vacuum and resuspended in 100 µl Urea buffer (50 mM Tris-Cl pH 7.5, 5 mM EDTA, 6 M Urea, 1% SDS). 200 µl 0.5 mm glass beads were added to the tubes and the cells were lysed in a bead beater 5 times for 45 sec with 1 min on ice in between. The extracts were incubated at 65°C for 10 min and centrifuged at 14000 rpm for 10 min before the addition of 200 µl of 2x Laemmli buffer. Samples were boiled for 5 min and separated on 8% SDS-PAGE gel. The proteins were visualized by ECL and imaged using a LAS 4000 instrument (GE).

Telomere length analysis by Southern blotting

Genomic DNA was prepared from log-phase cultures in YES rich media with NucleoSpin Tissue kit (MACHEREY-NAGEL) according to instructions from the manufacturer. Briefly, cells were spun for 5 min at 3000 rpm at 4°C and washed with 1 ml 10 mM EDTA, pH 8. Cell pellets were then resuspended in 600 µl sorbitol buffer (1.2 M sorbitol, 10 mM CaCl2, 0.1 M Tris/HCl pH 7.5, 35 mM β-mercaptoethanol) with 10 µg zymolase and incubated at 30°C for 30 min. Next, cells were spun for 10 min at 2,000g and supernatant was aspirated. Cell pellets were lysed in 180 µl Buffer T1 and 25 µL Proteinase K solution for 3 hrs at 56°C. Then 200 µl Buffer B3 was added and samples were incubated at 70°C for 12 min. DNA was precipitated by addition of 210 µl pure ethanol. The resuspended pellet was then transferred to the spin column, centrifuged for 1 min at 11000g and washed first with 500 µl Buffer BW followed by 600 µl Buffer B5. Lastly, DNA was eluted with 50 µl pre-warmed (70°C) Buffer BE.

For telomere length analysis, 1 µg of genomic DNA was digested with EcoRI-HF (NEB) overnight and gel electrophoresis was carried out on 1% agarose gels. Overnight transfer was in alkaline solution (0.4 M NaOH) onto nylon membrane (Roche). For hybridisation, the membrane was incubated in Church hybridisation buffer (0.5 M Sodium phosphate, pH 7.2, 7 % SDS) for 1 hr at 65°C. Radioactive labelling of a telomeric probe was from a cloned restriction fragment (Amersham Megaprime DNA Labelling System). The probe was purified (Illustra Microspin G-50 Columns), heat-denatured, and added directly into hybridisation tube for overnight incubation at 65°C. The membrane was then washed in 50 ml pre-warmed Church wash solution (40 mM

Sodium phosphate, pH7.2, 1% SDS) at 65°C for 5 min, followed by 20 min twice. Finally, the signals were visualized using a Fujifilm PhosphorImager Scanner (Molecular Dynamics).

Chromatin Immuno-precipitation

ChIP was performed as described previously [2, 3]. 50 ml of log-phase cells were crosslinked with 1% formaldehyde for 15 min at RT. The cells were washed in cold HBS (50 mM HEPES pH 7.5, 140 mM NaCl) and resuspended in ChIP lysis buffer (50 mM HEPES pH 7.5, 140 mM NaCl, 1 mM EDTA, 1% IGEPAL CA-630, 0.1% sodium deoxycholate, 1 mM PMSF, Roche Complete Mini protease inhibitors). An equal volume of silica beads was added and the cells were broken in a MiniBeadBeater for 3 min. The lysate was recovered and centrifuged for 30 min at full speed at 4°C. The pellet was resuspended in 600 μl ChIP lysis buffer and sonicated with a Bioruptor. Samples were centrifuged and the supernatant was recovered. About 3% of the supernatant was kept as whole-cell extract and the rest was used for the immunoprecipitation. 1 μg of the required antibody was added to the samples that were then rotated for 1 hr at 4°C. 40 μl of Dynabeads ProteinG (Invitrogen) per sample were washed thrice with lysis buffer and added to the samples. The samples were incubated for 2 additional hrs at 4°C. Beads were washed twice in SDS buffer (50mM HEPES, 1mM EDTA, 140 mM NaCl, 0.03% SDS), once in high salt buffer (50 mM HEPES, 1 mM EDTA, 1.0 M NaCl), once in T/L solution (20 mM Tris-Cl pH 7.5, 250 mM LiCl, 1 mM EDTA, 0.5% IGEPAL-CA630, 0.5% sodium deoxycholate), twice in T/E solution (20 mM Tris-Cl pH 7.5, 0.1 mM EDTA). The beads were resuspended in TES (20 mM Tris-Cl pH 7.5, 0.1 mM EDTA, 1% SDS) and heated at 65°C for 2 min. Supernatants were reverse cross-linked at 65°C overnight and the DNA was purified using a Qiagen PCR Purification kit (eluted with 0.5x Quiagen Elution Buffer).

WCEs (10 μl) and IPs (40 μl) were taken to a final volume of 40 μl, 10 μl of denaturation buffer (1.5 M NaOH, 3 M NaCl) was added, and samples were incubated for 10 min at room temperature. 130 μl of dilution buffer (0.1x SSC, 0.125 M NaCl) were added and samples were incubated for 5 min on ice. The DNA was vacuum-spotted using a S&S Minifold I Dot Blot system onto a nylon membrane (Roche) pre-equilibrated with dilution buffer. The membrane was crosslinked, washed in neutralization buffer (0.5 M Tris-Cl pH 7.5, 0.5 M NaCl) and probed with a

P^{32}-labelled telomere probe (same as for Southerns). The signals were visualized using a Fujifilm PhosphorImager Scanner (Molecular Dynamics) and quantified using ImageQuant TL (GE Healthcare). In order to correct for overall differences in the efficiency of the immuno-precipitation between experiments, each experiment was normalized against its highest peak value.

Two-hybrid analysis

Yeast two hybrid assays were performed by co-transforming GBD (Gal4 DNA Binding Domain) and GAD (Gal4 Activating Domain) plasmids in various combinations in the PJ69-4A budding yeast strain (*MATa trp1-901 leu2-3,112 ura3-52 his3-200 gal4 gal80 LYS2::GAL1-HIS3 GAL2-ADE2 met2::GAL7-lacZ*). Transformants were screened for interaction by spotting 5-fold dilutions on selective synthetic complete drop-out media (SC-TRP-LEU-HIS and SC-TRP-LEU-HIS-ADE).

Generation of the *stn1-75* allele

A wild-type strain was transformed with a BstBI-HindIII fragment from pAB1447 to make a strain containing a loxP-Stn1-Ura4-loxM3 cassette integrated at the *stn1* locus [4]. In this strain the cassette replaces the endogenous *stn1* open reading frame with an identical one but devoid of introns. The intronless *stn1* orf is under control of the endogenous *stn1* promoter but is preceded by a loxP site. Strains bearing this cassette (*stn1-LP* allele) maintain telomeres, unlike a null *stn1* allele, but these are of increased length, presumably due to altered expression levels. This strain was then transformed with BglII-linearized pAB1535 to delete *rqh1+* in order to prevent circularization of chromosomes upon telomere loss [5], and subsequently with pAB1448, for thiamine-controlled expression of Cre recombinase. Error-prone PCR was used to amplify pAB1606 to generate PCR products containing a mutagenised loxP-Stn1-loxM3 cassette to be used for transformation of the *stn1-LP* strain. Transformants were plated on YNG–Leu plates without thiamine, incubated at 25°C for 2 days, before replica plating onto YES + 5-FOA and further incubated at 25°C. Once colonies were visible they were replica plated to two fresh 5-FOA plates, one of which was incubated at 25°C, the other at 36°C for another 2-3 days to identify temperature-sensitive alleles.

References

1. Ulrich HD, Davies AA (2009) In vivo detection and characterization of sumoylation targets in *Saccharomyces cerevisiae*.. *Methods Mol Biol* **497:** 81-103

2. Bianchi A, Shore D (2007) Early replication of short telomeres in budding yeast. *Cell* **128:** 1051-1062

3. Jain D, Hebden AK, Nakamura TM, Miller KM, Cooper JP (2010) HAATI survivors replace canonical telomeres with blocks of generic heterochromatin. *Nature* **467:** 223-227

4. Watson AT, Garcia V, Bone N, Carr AM, Armstrong J (2008) Gene tagging and gene replacement using recombinase-mediated cassette exchange in *Schizosaccharomyces pombe*. *Gene* **407:** 63-74

5. Nanbu T, Takahashi K, Murray JM, Hirata N, Ukimori S, Kanke M, Masukata H, Yukawa M, Tsuchiya E, Ueno M (2013) Fission Yeast RecQ Helicase Rqh1 Is Required for the Maintenance of Circular Chromosomes. *Mol Cell Biol* **33:** 1175-1187
