# Supplementary material for: Tpz1TPP1 SUMOylation reveals evolutionary conservation of SUMO-dependent Stn1 telomere association
Source: EMBO Rep. 2014 Jun 12;15(8):871–7. doi: 10.15252/embr.201438919 (PMC4197044; doi:10.15252/embr.201438919)
Supplement: Supplementary file 4 [file embr0015-0871-sd4.pdf]

EMBO
reports

Manuscript EMBO-2014-38919

Tpz1TPP1 SUMOylation reveals evolutionary conservation of SUMOdependent Stn1 telomere association

Mansi Garg, Resham L. Gurung, Sahar Mansoubi, Jubed O. Ahmed, Anoushka Davé, Felicity Z. Watts and Alessandro Bianchi

Corresponding author: Alessandro Bianchi, University of Sussex

Review timeline:

Submission date:	15 April 2014
Editorial Decision:	23 April 2014
Revision received:	14 May 2014
Editorial Decision:	19 May 2014
Accepted:	19 May 2014

Transaction Report:

(Note: With the exception of the correction of typographical or spelling errors that could be a source of ambiguity, letters and reports are not edited. The original formatting of letters and referee reports may not be reflected in this compilation.)

Editor: Nonia Pariente

No Peer Review Process File is available with this article, as the authors have chosen not to make the review process public in this case.

© European Molecular Biology Organization
